# Metabolomics Analysis of the Renal Cortex in Rats With Acute Kidney Injury Induced by Sepsis

**DOI:** 10.3389/fmolb.2019.00152

**Published:** 2019-12-20

**Authors:** Feng Ping, Yong Guo, Yongmei Cao, Jiawei Shang, Sijia Yao, Junfeng Zhang, Yingchuan Li

**Affiliations:** ^1^Department of Anesthesiology and Critical Care Medicine, Shanghai Jiao Tong University Affiliated Sixth People's Hospital, Shanghai, China; ^2^Section of Nutrition Research, Division of Digestive Diseases, Department of Metabolism, Digestion and Reproduction, Faculty of Medicine, Imperial College London, London, United Kingdom

**Keywords:** renal cortex, metabolomics, acute kidney injury, sepsis, LPS

## Abstract

Sepsis-induced acute kidney injury (AKI) can increase the mortality of critically ill patients and the incidence of chronic kidney disease in critically ill survivors. The main goal was to investigate the possible link between metabolic changes and sepsis-induced AKI development. The experimental animal model of sepsis-induced AKI was established by intraperitoneal injection of lipopolysaccharide in rats. Non-targeted metabolic screening of the renal cortex in the control and sepsis-induced AKI groups was carried out based on gas chromatography coupled with quadrupole time-of-flight mass spectrometry (GC-TOFMS) technology. The data between the two groups were analyzed by combining univariate and multivariate statistical methods, and the metabolites associated with AKI in rats with sepsis were screened. By examining the Kyoto Encyclopedia of Genes and Genomes (KEGG) database, altered metabolic pathways associated with acute renal injury in sepsis were identified. The cross validated scores plot of orthogonal partial least squares discriminant analysis (OPLS-DA) showed a distinct separation trend between the model and control groups in the profile of renal cortex metabolites, indicating a significant change in endogenous metabolites in the rat renal cortex. Further analysis and screening showed that 26 different metabolites were identified in the renal cortex between the two groups, mainly involving taurine and hypotaurine metabolism, pantothenic acid and CoA biosynthesis, phenylalanine metabolism, and other metabolic pathways. The metabolic disorders of taurine, pantothenic acid, and phenylalanine in the renal cortex are related to the development of acute renal injury in sepsis. Correcting these metabolic disorders is expected to prevent and treat sepsis-induced AKI.

## Introduction

Sepsis and septic shock are important risk factors for acute kidney injury (AKI). An estimated 64% of patients with sepsis have AKI (Bagshaw et al., 2009). AKI is a major cause of morbidity and mortality in critically ill patients and can progress to chronic kidney disease and dialysis dependency (Clermont et al., 2002). Despite its prevalence and associated mortality, our understanding of the pathogenesis of septic-induced AKI is limited. Renal vasoconstriction and reduced renal blood flow leading to renal ischemia, as well as the inflammation and apoptosis of renal tubular cells, contribute to kidney injury in sepsis. Evidence demonstrates that the depletion of ATP and activation of multiple enzyme systems, including proteases, nitric oxide synthases, and phospholipases, are responsible for cell and organ dysfunction and eventually result in cell death in acute injured kidneys (Kribben et al., 1994; Edelstein et al., 1997; Goligorsky et al., 2002). Since the kidney is the major organ for excreting metabolic end-products, its injury can cause changes in the renal metabolic profile, and understanding the influence of AKI on kidney function will shed light on new potential diagnostic markers and therapeutic targets in AKI (Makris and Kafkas, 2012; Wei et al., 2014).

Metabolomics is an efficient technique for detecting perturbations in the metabolome and for providing detailed information on the biochemical phenotype of an organism in a healthy or pathological state (Patti et al., 2012). Metabolomics can be used to study chemical fingerprints of cellular processes and allow dynamic monitoring of the biochemical alterations induced by various environmental and pathological stimuli (Nicholson and Lindon, 2008). Thus, metabolic profiling combined with chemometric analysis has the ability to measure global alterations in metabolism within tissues or biofluids that precede conventional biochemical and pathological changes. Several studies have utilized metabolomics to study AKI induced by toxins and antibiotics (Weiss and Kim, 2012), and recently, a serum metabolomics approach has been used in a pilot study to discover novel serum biomarkers of AKI (Sun et al., 2012).

In the current study, the metabolomic changes in the renal cortex in a rat model with LPS-induced AKI were studied by gas chromatography time flight mass spectrometry (GC-TOFMS) to identify altered metabolites and metabolic pathways associated with the development of sepsis-induced AKI, to better understand the pathogenesis of sepsis-induced AKI and to provide new insights into the development of early diagnosis and treatments for sepsis-induced AKI.

## Materials and Methods

### Animal Model Preparation and Grouping

SPF male Sprague-Dawley (SD) rats weighing 200–250 g were purchased from the Department of Laboratory Animal Science, Shanghai Jiaotong University. Thirty SD rats were randomly divided into a control group, LPS 2 h group and LPS 6 h group with eight rats in each group. In general, eight or more tissue samples in each rat group can meet the statistical efficiency in metabolomics analysis. Control group: intraperitoneal injection of PBS was performed; LPS 2 h group: samples were taken 2 h after intraperitoneal injection of LPS; LPS 6 h group: samples were taken 6 h after intraperitoneal injection of LPS. After 1 week of feeding without intervention, rats in the LPS 2 h group and LPS 6 h group were injected with LPS (dose of 5 mg/kg, concentration of 5 mg/ml, dissolved in PBS), while rats in the control group were injected with the same amount of PBS solution. Sample collection: Rats were anesthetized with pentobarbital sodium at 2 and 6 h after LPS treatment. Inferior vena cava blood and left kidney tissue were obtained from the LPS 2 h group, LPS 6 h group and control group. The blood was placed in a centrifugal tube for 30 min at room temperature and then centrifuged for 10 min at 3,000 r/min. The serum was harvested and divided into two parts, one for serum biochemical detection and the other for metabolomics detection. These serum samples were all stored at −80°C in a refrigerator. The left renal cortex tissue was fixed in 4% paraformaldehyde and stained with HE and PAS (Periodic Acid-Schiff stain). All biosecurity and safety procedures are carried out in accordance with the regulations of Shanghai Jiao Tong University.

### Reagents and Instruments

LPS (Escherichia coli 055: B5), methoxyamine hydrochloride, fatty acid methyl ester (C7-C30, FAMEs), anhydrous pyridine, and anhydrous sodium sulfate were purchased from Sigma-Aldrich (St. Louis, MO, USA). PBS, derivatization reagent MSTFA (containing 1% TMCS), methanol (Optima LC-MS), and n-ethane were purchased from Thermo Fisher (Fair Lawn, NJ, USA). Dichloromethane, chloroform and acetone were purchased from the China National Pharmaceutical Group Corporation, Peking, China. Chloral hydrate powder and paraformaldehyde were purchased from Sinopharm Chemical Reagent Co., Ltd, Shanghai, China. The AXSYM automatic immunoassay analyzer for liver and kidney function tests was purchased from Abbott Laboratories, Illinois, USA. Experimental ultrapure water was prepared from a Millipore Reference ultrapure water system (Billerica, MA, USA) equipped with a liquid chromatography coupled 0.22 μm filter. A GC-TOFMS spectrometer was purchased from (LECO Corp., St. Joseph, MI, USA).

### HE Staining of Paraffin Sections

Fresh tissue was fixed in 4% paraformaldehyde for more than 24 h. The tissue was removed from the fixing fluid and trimmed with a scalpel in the fume cabinet. The trimmed tissue and the corresponding label were placed in the dehydration box. The dehydration box was placed in a hanging basket and dehydrated in a dewatering machine with an alcohol gradient as follows: 75% alcohol for 4 h, 85% alcohol for 2 h, 90% alcohol for 2 h, 95% alcohol for 1 h, absolute alcohol I for 30 min, absolute alcohol II for 30 min, alcohol benzene for 5–10 min, xylene I for 5–10 min, xylene II for 5–10 min, wax I for 1 h, wax II for 1 h, and wax III for 1 h. The wax-soaked tissue was embedded in the embedding machine. The melted wax was first put into the embedding frame. Before the wax solidified, the tissues were removed from the dehydration box and placed into the embedding frame according to the requirements of the embedding surface and affixed with corresponding labels. The embedding frame was cooling on the freezing table at −20°C. After the wax solidified, the wax block was removed from the embedding frame, and the wax block was trimmed. The trimmed wax blocks were placed on the paraffin slicing machine and sliced. The thickness of the slices was 4 μ. Slices were placed in the spreader to float in water (40°C) to flatten the tissue. The tissue was picked up with slides and baked in a 60°C oven. After the wax was dried and heated, slices were removed and stored at room temperature for use. The slices were sequentially placed in xylene I for 20 min, xylene II for 20 min, anhydrous ethanol I for 10 min, anhydrous ethanol II for 10 min, 95% ethanol for 5 min, 90% ethanol for 5 min, 80% ethanol for 5 min, 70% ethanol for 5 min, and distilled water for washing. Slices were stained with Harris hematoxylin for 3–8 min, washed with tap water, differentiated with 1% hydrochloric acid and alcohol for several seconds, washed with tap water, returned to blue with 0.6% ammonia water, and washed with running water. Slices were stained in eosin staining solution for 1–3 min. The slices were dehydrated and rendered transparent by sequentially placing them into 95% alcohol I for 5 min, 95% alcohol II for 5 min, absolute alcohol I for 5 min, absolute alcohol II for 5 min, xylene I for 5 min, and xylene II for 5 min. The slices were removed from xylene, allowed to dry slightly, and then sealed with neutral gum. The staining results showed that the nucleus was blue and the cytoplasm was red.

### Serum Biochemical Detection

The serum liver and kidney functions of rats in the control group, LPS 2 h group and LPS 6 h group were measured by an AXSYM automatic immunoassay.

### Metabolomics Analysis

Untargeted metabolomics profiling was conducted on the XploreMET platform (Metabo-Profile Biotechnology, Shanghai, China) according to previously published methods with minor modifications (Qiu et al., 2009; Ni et al., 2012). Briefly, the 50 mg frozen samples that were harvested and stored in microcentrifuge tube, were mixed with 25 mg of pre-chilled zirconium oxide beads and 10 μL of internal standard. Each aliquot of 50 μL of 50% pre-chilled methanol were added for automated homogenization. After centrifugation at 14,000 g and 4°C for 20 min (Microfuge 20R, USA), the supernatant was transferred to an autosampler vial (Agilent Technologies, USA). Each aliquot of 175 μL of pre-chilled methanol/chloroform (v/v = 3/1) was added to the residue for the second extraction. After centrifugation at 14,000 g and 4°C for 20 min, each 200 μL supernatant was transferred to an autosampler vial. The remaining supernatant from each sample was pooled to make quality control samples. All the samples in autosampler vials were evaporated briefly to remove chloroform using a CentriVap vacuum concentrator (Labconco, USA), and further lyophilized with a FreeZone freeze dryer equipped with a stopping tray dryer. The sample derivatization and injection were performed by a robotic multipurpose sample MPS2 with dual heads (Gerstel, Germany). Briefly, the dried sample was derivatized with 50 μL of methoxyamine (20 mg/mL in pyridine) at 30°C for 2 h, followed by the addition of 50 μL of MSTFA (1% TMCS) containing FAMEs as retention indices at 37.5°C for 1 h. The derivatized samples were injected for analysis after derivatization. Each specimen was introduced for GC-TOFMS analysis on a time-of-flight mass spectrometry (GC-TOF/MS) system (Pegasus HT, Leco Corp., St. MO, USA) with an Agilent 7890B gas chromatograph. Gas chromatography settings: The chromatographic column was Rxi-5MS (30 m_250 μm_0.25 μm) with an injection volume of 1.0 μl. The carrier gas was helium, the column velocity was 1.0 mL/min, the inlet temperature was 270°C, and the transmission interface temperature was 270°C. The temperature programmed conditions of the oven were as follows: 80°C was maintained for 2 min, increased to 300°C by 12 C/min, and increased to 320°C by 40°C/min after 4.5 min, which was maintained for 1 min. Mass spectrometry settings: The ionization mode was electron collision, electron energy −70 eV, detector voltage −1,450 V, source temperature 220°C, acquisition rate 25 spectra per second, and mass range 50–550 Da.

Self-developed metabolomics software Xplore MET was used to automatically compare the retention index of deconvoluted peak signals and mass spectrometry fragments with JIALIB, which has the largest database of endogenous metabolites based on silylation-derived gas chromatography-mass spectrometry (including more than 1,500 endogenous metabolites thus far). The original data generated by GC-TOFMS were automatically exported to Xplore MET through ChromaTOF software for baseline smoothing and correction, deconvolution, extraction, and alignment of original chromatographic peak signals, retention index correction, metabolite identification, and data preprocessing (normalization and standardization).

The specific aims of our metabolomics analysis included the following: providing untargeted profiling of endogenous small-molecule metabolites in the study samples, annotating metabolites using a comprehensive proprietary mammalian metabolite database (Metabo-Profile Biotechnology, Shanghai, China), calculating the ratios of the two adjacent metabolites from known metabolic relation network (KEGG), obtaining metabolic profile differences between groups using both multivariate and univariate statistical tools, and performing metabolic pathway enrichment analysis (MPEA) to identify the most relevant metabolic pathways.

### Statistical Analysis

The raw data generated by GC-TOF/MS were processed using XploreMET for automated baseline denosing and smoothing, peak picking and deconvultion, creating reference database from the pooled QC samples, metabolite signal alignment, missing value correction and imputation, and QC correction. Each data set was transformed into comparable data vectors for statistical analysis. All measurements were mean-centered and scaled by the standard deviation of the observed measurements.

SPSS 19.0 software was used for statistical analysis, and GraphPad Prism 6.0 software was used for drawing. An independent sample *t*-test, *P* < 0.05, indicated that the difference was statistically significant. Xplore MET software has the functions of data processing, interpretation, and visualization. Data automatically imported into Xplore MET can be used for multidimensional statistical analysis, such as principal component analysis (PCA) and orthogonal projections to latent structures discriminant analysis (OPLS-DA), as well as one-dimensional statistical analysis, such as Student's *t*-test. Thus, the model can be evaluated according to the relevant parameters.

## Results

### The Effect of Sepsis on Renal Function and Liver Function

**Figure 1:**
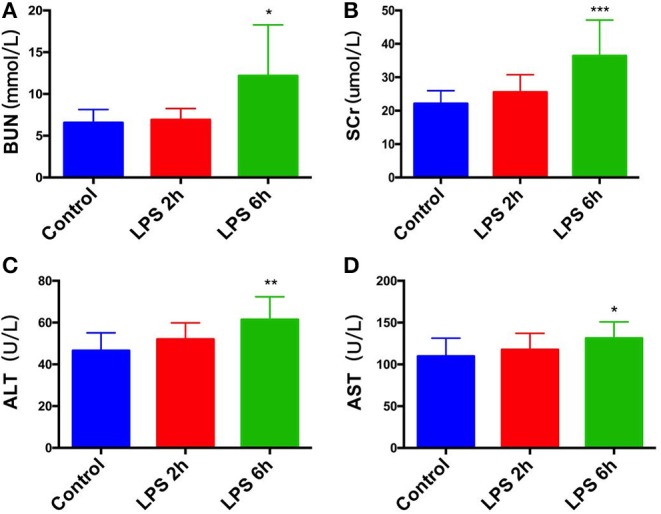
Effect of PBS or LPS treatment on renal function and liver function in rats. **(A)** Blood urea nitrogen (BUN) levels in different groups, *p < 0.05. **(B)** Serum creatinine (SCr) levels in different groups, ***p < 0.001. **(C)** Alanine aminotransferase (ALT) levels in different groups, **p < 0.01. **(D)** glutamic oxaloacetic aminotransferase (AST) levels in different groups, *p < 0.05.

Compared with the control group, the LPS 2 h group showed no significant changes in serum urea nitrogen (Figure 1A), creatinine (Figure 1B), alanine aminotransferase (Figure 1C), and glutamic oxaloacetic aminotransferase (Figure 1D) (*p* > 0.05). In contrast, the LPS 6 h group showed significant increases in serum urea nitrogen, creatinine, alanine aminotransferase, and glutamic oxaloacetic aminotransferase (*p* < 0.05, *p* < 0.001, *p* < 0.01, *p* < 0.05, respectively). The result is shown in Figure 1.

### Pathological Changes in Kidney Tissue Caused by Sepsis

**Figure 2:**
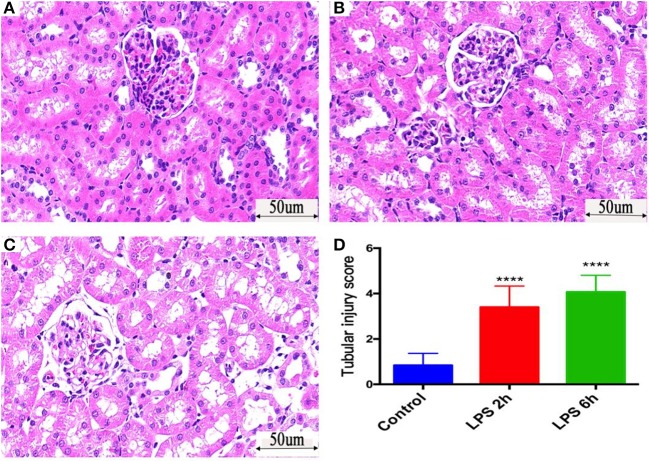
Histological assessment of injury in the kidneys of PBS- or LPS-treated rats by HE-based staining. **(A)** HE staining of kidney tissue in the control group. **(B)** HE staining of kidney tissue in the LPS 2 h group. **(C)** HE staining of kidney tissue in the LPS 6 h group. **(D)** The renal tubular injury scores in different groups, ****p < 0.0001.

HE staining: Compared with those in the control group (Figure 2A), the epithelial cells of renal tubules in the LPS 2 h group (Figure 2B) and LPS 6 h group (Figure 2C) showed vacuolar degeneration, hair brush margin shedding, and tubular lumen enlargement. Additionally, necrosis and shedding of epithelial cells and tubular formation were observed. The renal tubular injury scores of three groups of HE-stained kidney tissues were evaluated (Coulson et al., 2005). Figure 2D is the statistical result of three groups of renal injury scores. The renal injury scores of rats in the LPS 2 h group and LPS 6 h group were significantly higher than those in the control group (*p* < 0.0001).

**Figure 3:**
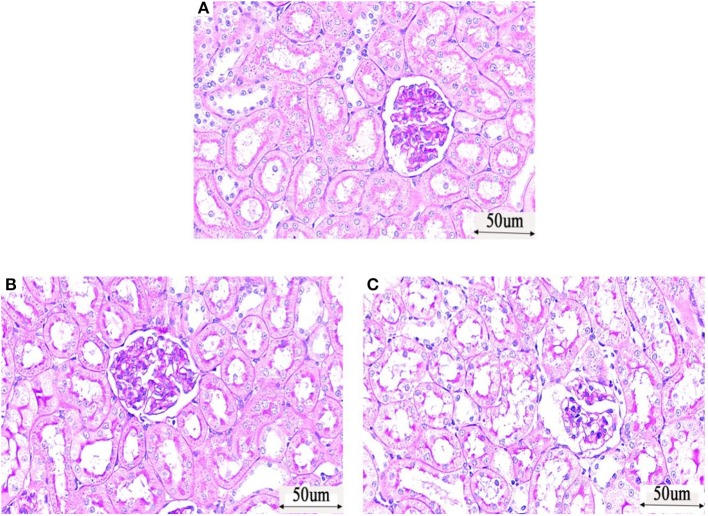
Histological assessment of injury in the kidneys of PBS- or LPS-treated rats by PAS (Periodic Acid-Schiff stain)—based staining. **(A)** PAS staining of kidney tissue in the control group. **(B)** PAS staining of kidney tissue in the LPS 2 h group. **(C)** PAS staining of kidney tissue in the LPS 6 h group.

PAS (Periodic Acid-Schiff stain) staining: The glomeruli, tubules and interstitial structure of the control group (Figure 3A) were normal, and the basement membrane was well-colored. No abnormal changes, such as inflammatory cell infiltration and fibrosis, were observed. The tubular lumen of rats in the LPS 2 h group (Figure 3B) and LPS 6 h group (Figure 3C) was obviously dilated, the basement membrane of tubular epithelial cells was not continuous, and the epithelial cells were irregular and different in size. The arrangement of tubular epithelial cells was disordered in these groups compared with that in the control group, and cell abscission was observed.

According to the above results, renal pathological damage formed 2 h after LPS administration, while the traditional renal function indicators SCr and BUN showed no significant changes, which proves that SCr and BUN are not sufficiently sensitive early diagnostic indicators of sepsis renal injury. Liver function was normal at this time; thus, metabolites that change significantly 2 h after LPS administration have potential as early diagnostic markers of sepsis-induced AKI.

### Metabolomics Analysis of the Renal Cortex in Rats With AKI

**Figure 4:**
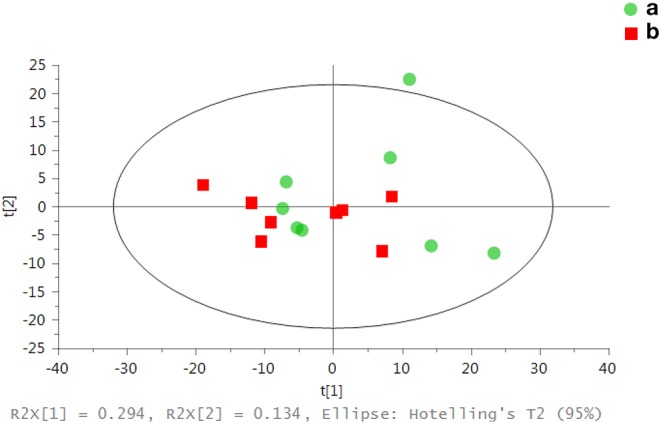
Assessment of metabolic differences in renal samples from both groups by PCA scores: each point in the figure represents the overall metabolic profile of a serum sample. (a) represents the renal cortex of the control group. (b) represents the renal cortex of the LPS 2 h group.

**Figure 5:**
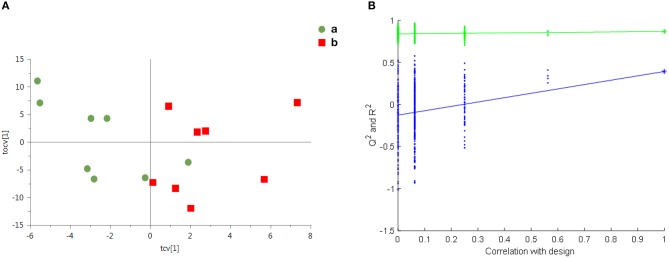
Overall metabolite profile difference between the two groups: **(A)** the cross validated scores plot of OPLS-DA (Q2Y = 0.49; R2X = 39%; One predictive component and one orthogonal component); **(B)** 500 permutation times (*p* = 0.04).

Model validity evaluation of renal cortex samples: According to the results of PCA in Figure 4, the renal cortex of the control group (Figure 4a) and the renal cortex of the LPS 2 h group (Figure 4b) did not exceed the limit, and there was no significant difference in the metabolic profile between the samples in each group. Figure 5A shows the cross validated scores plot OPLS-DA model (Q2Y = 0.49; R2X = 39%) of renal cortex samples from the control group (A) and LPS 2 h group (B). Figure 5B shows the permutation test plot with R2 (Permutation test with 500 times, *p* = 0.04). Therefore, the cross validated scores of OPLS-DA model of the renal cortex in both groups was effective and had good prediction ability (Figures 5A,B).

**Figure 6:**
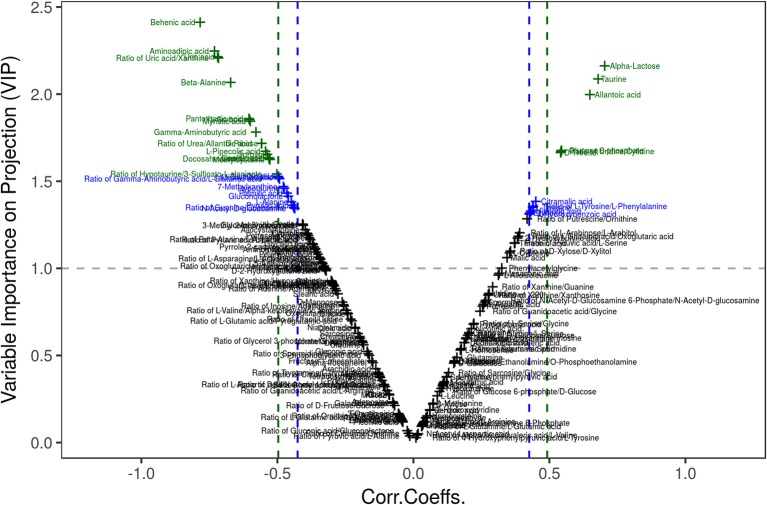
Visualization of differential metabolite profiles using Vplot. A volcano plot has been proposed for reliable metabolite marker selection because it combines the strength of both contribution (variable importance in projection, VIP) and variable reliability (correlation coefficients, Corr.) from the same OPLS-DA model.

Multivariate statistical analysis: There was no significant difference in metabolic profile between the samples in each group by PCA. The differential metabolites were obtained using a multicriteria assessment in an OPLS-DA model. Thus, a volcano plot (Figure 6) has been proposed for reliable metabolite marker selection because it combines the strength of both contribution (variable importance in projection, VIP) and variable reliability (correlation coefficients, Corr.) from the same OPLS-DA model. The value of the VIP score, which was >1, is the typical rule for selecting relevant variables (Table 1). With a significance level of 0.05, a corresponding Corr. value was used as a cutoff value to select the variables that were most correlated with the very first predictive component. The list of 26 differential metabolites is provided in Table 1.

**Table 1:** Differential metabolites between the groups using VIP ≥ 1 and Corr. Coeffs. at *p* < 0.05.

**Class**	**Name**	**HMDBID**	**KeggID**	**VIP**	**Corr. Coeffs**.	***P***
Amino Acid	Allantoic acid	HMDB01209	C00499	2.0	0.65	6.5e-03
	Methylcysteine	HMDB02108	NA	1.6	−0.53	3.5e-02
	Ratio of urea/allantoic acid	HMDB00294/HMDB01209	C00086/C00499	1.7	−0.56	2.5e-02
	Gamma-aminobutyric acid	HMDB00112	C00334	1.8	−0.58	1.9e-02
	Beta-alanine	HMDB00056	C00099	2.1	−0.67	4.3e-03
	Aminoadipic acid	HMDB00510	C00956	2.2	−0.73	1.3e-03
Carbohydrates	Alpha-lactose	HMDB00186	C00243	2.2	0.7	2.4e-03
	Glucose 6-phosphate	HMDB01401	C00092	1.7	0.54	2.9e-02
	D-Threitol	HMDB04136	C16884	1.7	0.54	3.0e-02
	D-Xylitol	HMDB02917	C00379	1.5	−0.5	5.0e-02
	Sorbitol	HMDB00247	C00794	1.7	−0.54	3.2e-02
	D-Ribose	HMDB00283	C00121	1.7	−0.56	2.5e-02
	L-Sorbose	HMDB01266	C08356	1.9	−0.6	1.3e-02
Fatty Acids	Linoleic acid	HMDB00673	C01595	1.6	−0.53	3.4e-02
	Caproic acid	HMDB00535	C01585	1.6	−0.53	3.5e-02
	Docosahexaenoic acid	HMDB02183	C06429	1.6	−0.53	3.5e-02
	Myristic acid	HMDB00806	C06424	1.8	−0.6	1.4e-02
	Behenic acid	HMDB00944	C08281	2.4	−0.78	3.2e-04
Nucleotide	Ratio of uridine/cytidine	HMDB00296/HMDB00089	C00299/C00475	1.7	0.54	3e-02
Organic acids	Taurine	HMDB00251	C00245	2.1	0.68	3.8e-03
	Ratio of hypotaurine/3-sulfinato-L-alaninate	HMDB00965/HMDB60179	C00519/C00606	1.5	−0.5	4.8e-02
	Glutaric acid	HMDB00661	C00489	1.5	−0.5	5.0e-02
	L-pipecolic acid	HMDB00716	C00408	1.7	−0.54	3.0e-02
	Uric acid	HMDB00289	C00366	2.2	−0.72	1.6e-03
	Ratio of uric acid/xanthine	HMDB00289/HMDB00292	C00366/C00385	2.2	−0.72	1.8e-03
Vitamin	Pantothenic acid	HMDB00210	C00864	1.9	−0.6	1.3e-02

*VIP, variable importance in the projection; KEGG ID, ID in the Kyoto Encyclopedia of Genes and Genomes; HMDBID, ID in the Human Metabolome Database; Corr. Coeffs., correlation coefficient*.

**Figure 7:**
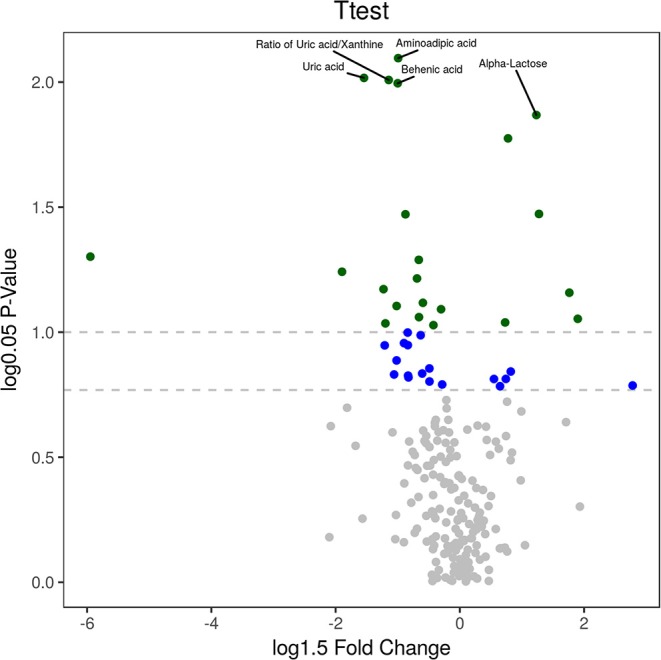
Visualization of differential metabolite profiles. An enhanced volcano plot showed the differential metabolites selected with the multicriteria assessment. The *p*-value together with log 1.5 (fold change, FC) were introduced with cutoff values of 0.05 and 0.01 for the *p*-value and 1.5 for log1.5 FC, respectively.

**Figure 8:**
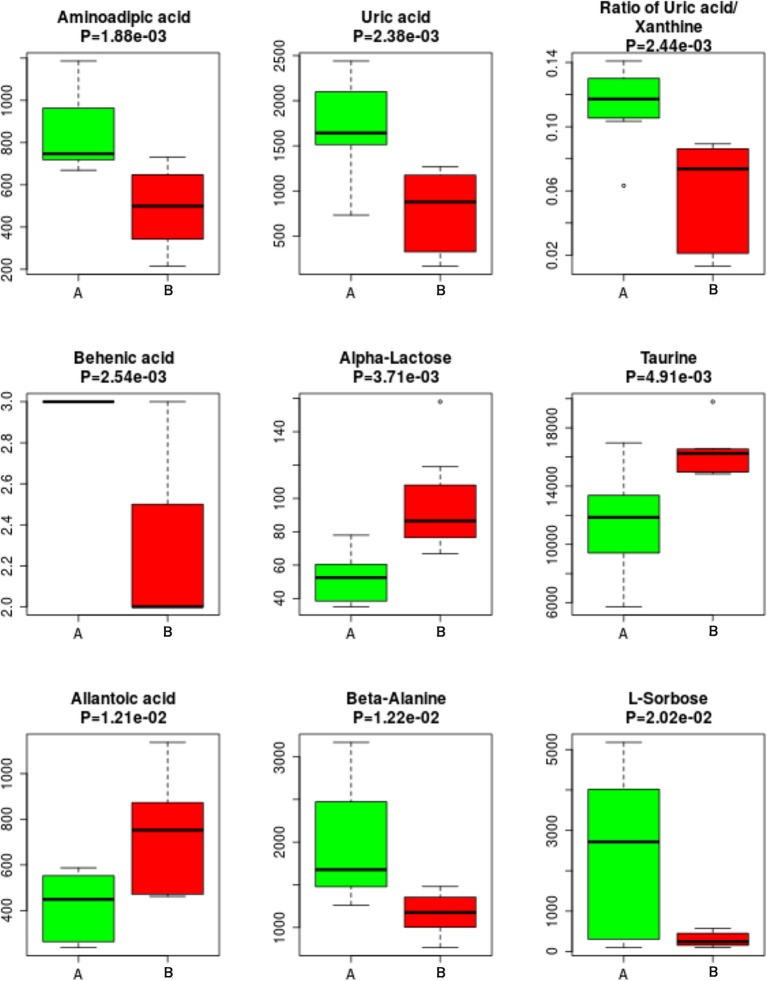
Top ranked differential metabolites between the two groups. The nine representative differential metabolites (top ranked) obtained from univariate statistical analysis are illustrated in this figure.

Univariate statistical analysis: The differential metabolites could also be obtained using univariate statistical analysis (Student's *t*-test), especially when the multivariate models failed to build reliable discriminant models under some conditions (i.e., non-homogeneous classes and large intraclass variability). An enhanced volcano plot showed the differential metabolites selected with the multicriteria assessment (Figure 7). The *p*-value together with log 1.5 (fold change, FC) were introduced with cutoff values of 0.05 and 0.01 for the *p*-value and 1.5 for log1.5 FC, respectively. The 23 differential metabolites obtained from univariate statistical analysis are summarized in Table 2. The nine representative differential metabolites (top ranked) obtained from univariate statistical analysis are illustrated in Figure 8.

**Table 2:** Differential metabolites between the groups using univariate statistical analysis.

**Class**	**Name**	**HMDBID**	**KeggID**	***p***	**FC**
Amino acid	Aminoadipic acid	HMDB00510	C00956	1.9e-03	0.7
	Allantoic acid	HMDB01209	C00499	1.2e-02	1.7
	Beta-Alanine	HMDB00056	C00099	1.2e-02	0.7
	Ratio of urea/allantoic acid	HMDB00294/HMDB01209	C00086/C00499	3.0e-02	0.6
	Gamma-aminobutyric acid	HMDB00112	C00334	3.6e-02	0.7
	Methylcysteine	HMDB02108	NA	4.2e-02	0.8
Carbohydrates	Alpha-lactose	HMDB00186	C00243	3.7e-03	1.6
	L-Sorbose	HMDB01266	C08356	2.0e-02	0.1
	D-Ribose	HMDB00283	C00121	2.1e-02	0.8
	D-Threitol	HMDB04136	C16884	3.1e-02	2.0
	Sorbitol	HMDB00247	C00794	3.5e-02	0.8
	Glucose 6-phosphate	HMDB01401	C00092	4.3e-02	2.2
Fatty acids	Behenic acid	HMDB00944	C08281	2.5e-03	0.7
	Myristic acid	HMDB00806	C06424	2.4e-02	0.5
	Caproic acid	HMDB00535	C01585	3.8e-02	0.9
	Docosahexaenoic acid	HMDB02183	C06429	4.5e-02	0.6
	Linoleic acid	HMDB00673	C01595	4.6e-02	0.8
Nucleotide	Ratio of uridine/cytidine	HMDB00296/HMDB00089	C00299/C00475	4.4e-02	1.3
Organic Acids	Uric acid	HMDB00289	C00366	2.4e-03	0.5
	Ratio of uric acid/xanthine	HMDB00289/HMDB00292	C00366/C00385	2.4e-03	0.6
	Taurine	HMDB00251	C00245	4.9e-03	1.4
	L-Pipecolic acid	HMDB00716	C00408	5.0e-02	0.7
Vitamin	Pantothenic acid	HMDB00210	C00864	2.6e-02	0.8

*FC, fold change; FCs were calculated by comparing the average value for the LPS 2 h group to the average value for the control group; FC with a value greater than one indicates a higher level of the metabolite in the LPS 2 h group than that in the control group; an FC value less than one indicates a lower level in LPS 2 h group than that in control group; KEGG ID, ID in the Kyoto Encyclopedia of Genes and Genomes; HMDBID, ID in the Human Metabolome Database*.

**Figure 9:**
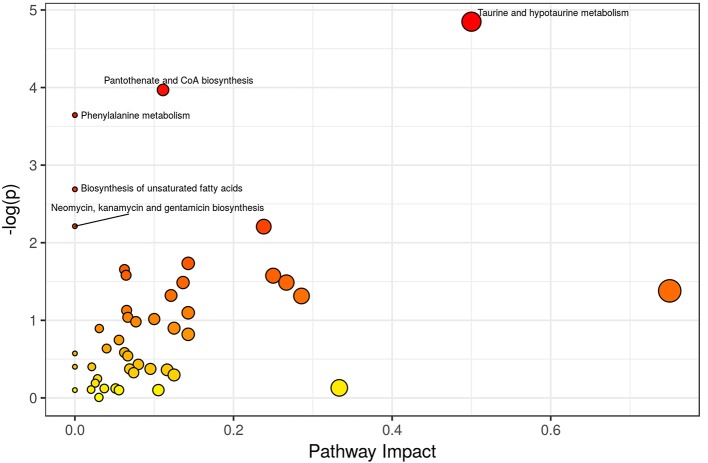
Overview of Pathway Analyses. Metabolic pathway enrichment analysis (MPEA) was performed to identify the most relevant metabolic pathways. The metabolic pathways most relevant to sepsis-induced AKI included taurine and hypotaurine metabolism, pantothenate and CoA biosynthesis, and phenylalanine metabolism.

Different metabolic pathways associated with sepsis-induced AKI: The MPEA results are summarized in Table 3 and Figure 9. Key metabolic pathways are highlighted with green (*p* ≤ 0.05 and 0.05 < *p* ≤ 0.1). The upregulated or downregulated metabolites are also provided in Table 3. The metabolic pathways most relevant to sepsis-induced AKI included taurine and hypotaurine metabolism, pantothenate and CoA biosynthesis, and phenylalanine metabolism.

**Table 3:** Differential metabolic pathway.

**Pathway name**	**P. hyper**	**Impact**	**Up**	**Down**
Taurine and hypotaurine metabolism	7.84e-03	0.50	Taurine; L-Cysteine	Hypotaurine
Pantothenate and CoA biosynthesis	1.89e-02	0.11	L-Cysteine	Beta-Alanine; Pantothenic acid; Uracil
Phenylalanine metabolism	2.61e-02	0.00	L-Tyrosine; Hippuric acid; Phenylacetylglycine	
Biosynthesis of unsaturated fatty acids	0.068	0.00		Palmitic acid; Linoleic acid; Arachidonic acid; Docosahexaenoic

*Hyer, hypergeometric test*.

## Discussion

In this study, the renal cortex of septic AKI rats and control rats was examined by GC-TOFMS. Renal pathological damage was formed 2 h after LPS was injected intraperitoneally, but there was no significant change in renal function indexes such as serum creatinine and urea nitrogen between the two groups. Through multivariate and univariate statistical analysis, 23 different metabolites were obtained in the renal cortex of the two groups, mainly involving taurine and hypotaurine metabolism, pantothenic acid and CoA biosynthesis, and phenylalanine metabolism.

Taurine is a semiessential amino acid that does not participate in protein synthesis. This amino acid plays many important physiological functions in mammals, such as osmotic regulation, nerve regulation, membrane stability and cell protection, antioxidant and anti-inflammatory effects, calcium homeostasis regulation (Oja and Saransaari, 2007; De Luca et al., 2015). Taurine therapy reportedly improves oxidative stress and histopathological kidney damage in rats with hypertension induced by the nitric oxide synthase inhibitor N-nitro-L-arginine-methyl ester (L-NAME) (Adedara et al., 2019). In the study of adriamycin-induced acute renal injury, taurine can improve the injury by inhibiting the production of inflammatory cytokines and the activity of caspase-3 and caspase-9 (Kim et al., 2017). In another model of I/R renal injury in mice, taurine levels in the plasma, renal cortex, and renal medulla did not change significantly at 2 and 48 h after reperfusion. After 1 week of reperfusion, taurine levels in the renal medulla increased significantly (*P* < 0.05) (Wei et al., 2014). In the rat model of renal I/R, taurine was given intravenously in advance, and the final serum creatinine level was much lower than that in control rats, which could significantly reduce renal injury (Michalk et al., 2003). Adding taurine to organ preservation solution can reduce tissue damage during hypoxia and reoxygenation and promote the recovery of energy metabolism in renal tubular cell transplantation model (LLC-PK1) cells (Wingenfeld et al., 1995). However, little research has been performed on taurine's renal protective role in septic kidney injury models. In the current study, taurine levels in the renal cortex of SD rats increased significantly 2 h after intraperitoneal injection of endotoxin LPS which may reflect the activation of renal self-protection. High levels of taurine have scavenging effects on elevated inflammatory factors, which may provide self-protection for the body after sepsis. The mechanism underlying the taurine-induced metabolic change and whether this change plays a protective role in the kidney require further experimental verification.

LPS challenge rapidly evokes excess generation of reactive oxygen species (ROS) that leads to complications of sepsis (Koyner et al., 2008). Oxidative stress is believed to play a key role in the development of AKI (Koyner et al., 2008). Studies have evaluated the mechanism of action of L-cysteine as a precursor for GSH synthesis in the context of its activity as an “antioxidant,” which could alleviate clinical symptoms of cystic fibrosis through cysteine-mediated disruption of disulfide cross-bridges in the glycoprotein matrix in mucus and protect against contrast-induced nephropathy and thrombosis (Rushworth and Megson, 2014). The protective effects of N-acetyl-cysteine on mitochondria and renal function could be related to its observed capacity to preserve the S-glutathionylation process and GSH levels in mitochondria (Aparicio-Trejo et al., 2019). Cysteine-rich protein 61 (Cyr61) reportedly increased in postischemic human kidney tissue, and this finding revealed that Cyr61 might replace Scr as an ultra-early new biomarker in AKI (Li et al., 2018). Our data suggest that L-cysteine is significantly elevated 2 h after intraperitoneal injection of endotoxin LPS, consistent with a previous study (Li et al., 2018). Elevated L-cysteine may also be a protective response of the kidney itself.

Beta-alanine is an isomer of alpha-alanine and belongs to alanine. This amino acid is a metabolite of uracil and 5-fluorouracil *in vivo*. Beta-alanine participates in the composition of vitamin B5 (pantothenic acid) and coenzyme A and synthesizes carnosine (beta-alanyl-L-histidine) *in vivo*. Carnosine can act as proton buffer (PKa = 6.83) (Davey, 1960), calcium regulator (Dutka and Lamb, 2004), antioxidant (Boldyrev, 2007), anti-aging factor (Gallant et al., 2000), protein glycosylation blocker (Hipkiss et al., 1995), wound healing agent, and protein cross-linking blocker (Nagai et al., 1986). Pantothenic acid mainly participates in the metabolism of sugar, lipid and protein in the form of CoenzymeA, which plays a role in transferring phenolic groups. In addition, pantothenic acid has an anti-lipid peroxidation effect. There are two possible mechanisms: (1) scavenging free radicals in the form of CoA to protect the cell plasma membrane from damage or (2) CoA helping cell repair by promoting phospholipid synthesis. Pantothenic acid can also increase the biosynthesis of glutathione, thereby slowing apoptosis and injury. Our results show that beta-alanine, pantothenic acid, and uracil decrease after LPS treatment, indicating that energy metabolism, pyrimidine metabolism, and antioxidant capacity are reduced in the renal cortex.

Normal kidneys are involved in amino acid metabolism. Phenylalanine hydroxylates to tyrosine, and glycine converts to serine. Studies on amino acid metabolism in patients with chronic renal failure (CRF) suggest that with the decrease in plasma free tyrosine and serine, the increases in phenylalanine/tyrosine ratio and glycine content are related to the severity of renal tissue damage. A metabolomics analysis showed that tryptophan, phenylalanine, lysophosphatidylcholine, creatinine, and canine urea were biomarkers in the serum of patients with CRF, which proved that the metabolism of amino acids and phospholipids was abnormal in CRF patients (Jia et al., 2008). Increases in acylcarnitines and amino acids (methionine, homocysteine, pyroglutamate, asymmetric dimethylarginine (ADMA), and phenylalanine) and a reduction in serum levels of arginine and several lysophosphatidylcholines were also observed in patients with AKI compared to those in healthy subjects (Sun et al., 2012). However, few metabolomics studies on the renal cortex of sepsis-induced AKI exist. Our data suggest that the elevation of L-tyrosine, hippuric acid, and phenylacetylglycine in the phenylalanine metabolic pathway in renal cortex after LPS treatment may be related to the elevation of phenylalanine and its metabolic derivatives in plasma under stress.

In summary, the altered metabolic pathways in the renal cortex are associated with sepsis-induced AKI, which includes taurine and hypotaurine metabolism, pantothenic acid and CoA biosynthesis, and phenylalanine metabolism. Strategies aiming to correct these metabolic disorders could improve outcomes and may provide new insights into the development of preventive treatments for sepsis-induced AKI.

## Data Availability Statement

All datasets generated for this study are included in the article/supplementary material.

## Ethics Statement

The animal study was reviewed and approved by Shanghai Jiao Tong University Affiliated Sixth People's Hospital.

## Author Contributions

YL designed the research. FP, YG, YC, JS, SY, and JZ conducted the research. YG and FP wrote the manuscript. All authors read and approved the final manuscript.

### Conflict of Interest

The authors declare that the research was conducted in the absence of any commercial or financial relationships that could be construed as a potential conflict of interest.

## References

[B1] Adedara I. A. Alake S. E. Olajide L. O. Adeyemo M. O. Ajibade T. O. Farombi E. O. (2019). Taurine ameliorates thyroid hypofunction and renal injury in L-NAME-induced hypertensive rats. Drug Res. 69, 83–92. 10.1055/a-0643-4604 29996172

[B2] Aparicio-Trejo O. E. Reyes-Fermin L. M. Briones-Herrera A. Tapia E. Leon-Contreras J. C. Hernandez-Pando R.. (2019). Protective effects of N-acetyl-cysteine in mitochondria bioenergetics, oxidative stress, dynamics and S-glutathionylation alterations in acute kidney damage induced by folic acid. Free Radic. Biol. Med. 130, 379–396. 10.1016/j.freeradbiomed.2018.11.005 30439416

[B3] Bagshaw S. M. Lapinsky S. Dial S. Arabi Y. Dodek P. Wood G.. (2009). Acute kidney injury in septic shock: clinical outcomes and impact of duration of hypotension prior to initiation of antimicrobial therapy. Intens. Care Med. 35, 871–881. 10.1007/s00134-008-1367-2 19066848

[B4] Boldyrev A. A. (2007). Carnosine and Oxidative Stress in Cells and Tissues. New York, NY: Nova Science Publishers.

[B5] Clermont G. Acker C. G. Angus D. C. Sirio C. A. Pinsky M. R. Johnson J. P. (2002). Renal failure in the ICU: Comparison of the impact of acute renal failure and end-stage renal disease on ICU outcomes. Kidney Int. 62, 986–996. 10.1046/j.1523-1755.2002.00509.x 12164882

[B6] Coulson M. T. Jablonski P. Howden B. O. Thomson N. M. Stein A. N. (2005). Beyond operational tolerance: effect of ischemic injury on development of chronic damage in renal grafts. Transplantation 80, 353–361. 10.1097/01.tp.0000168214.84417.7d 16082331

[B7] Davey C. L. (1960). The significance of carnosine and anserine in striated skeletal muscle. Arch. Biochem. Biophys. 89, 303–308. 10.1016/0003-9861(60)90059-x 13814256

[B8] De Luca A. Pierno S. Camerino D. C. (2015). Taurine: the appeal of a safe amino acid for skeletal muscle disorders. J. Transl. Med. 13:243. 10.1186/s12967-015-0610-1 26208967 PMC4513970

[B9] Dutka T. L. Lamb G. D. (2004). Effect of carnosine on excitation-contraction coupling in mechanically-skinned rat skeletal muscle. J. Muscle Res. Cell Motil. 25, 203–213. 10.1023/b:jure.0000038265.37022.c5 15467383

[B10] Edelstein C. L. Ling H. Schrier R. W. (1997). The nature of renal cell injury. Kidney Int. 51, 1341–1351. 10.1038/ki.1997.183 9150442

[B11] Gallant S. Semyonova M. Yuneva M. (2000). Carnosine as a potential anti-senescence drug. Biochem. Mosc. 65, 866–868. 10951107

[B12] Goligorsky M. S. Brodsky S. V. Noiri E. (2002). Nitric oxide in acute renal failure: NOS versus NOS. Kidney Int. 61, 855–861. 10.1046/j.1523-1755.2002.00233.x 11849438

[B13] Hipkiss A. R. Michaelis J. Syrris P. (1995). Non-enzymatic glycosylation of the dipeptide L-carnosine, a potential anti-protein-cross-linking agent. FEBS Lett. 371, 81–85. 10.1016/0014-5793(95)00849-5 7664889

[B14] Jia L. W. Chen J. Yin P. Y. Lu X. Xu G. W. (2008). Serum metabonomics study of chronic renal failure by ultra performance liquid chromatography coupled with Q-TOF mass spectrometry. Metabolomics 4, 183–189. 10.1007/s11306-008-0110-x

[B15] Kim Y. S. Sung S. H. Tang Y. J. Choi E. J. Choi Y. J. Hwang Y. J.. (2017). Protective effect of taurine on mice with doxorubicin-induced acute kidney injury. Taurine 975, 1191–1201. 10.1007/978-94-024-1079-2_95 28849533

[B16] Koyner J. L. Ali R. S. Murray P. T. (2008). Antioxidants - Do they have a place in the prevention or therapy of acute kidney injury? Nephron Exp. Nephrol. 109, E109–E117. 10.1159/000142935 18802373

[B17] Kribben A. Wieder E. D. Wetzels J. F. M. Yu L. Gengaro P. E. Burke T. J.. (1994). Evidence for role of cytosolic-free calcium in hypoxia-induced proximal tubule injury. J. Clin. Invest. 93, 1922–1929. 10.1172/JCI117183 8182125 PMC294299

[B18] Li C. Zhao L. Wang Y. Che L. Luan H. Luo C.. (2018). Cysteine-rich protein 61, a specific ultra-early biomarker in kidney ischemia/reperfusion injury. Nephrology. 24, 798–805. 10.1111/nep.13513 30328178

[B19] Makris K. Kafkas N. (2012). Neutrophil gelatinase-associated lipocalin in acute kidney injury. Adv. Clin. Chem. 58, 141–191. 10.1016/b978-0-12-394383-5.00012-6 22950345

[B20] Michalk D. V. Hoffmann B. Minor T. (2003). Taurine reduces renal ischemia/reperfusion injury in the rat. Adv. Exp. Med. Biol. 526, 49–56. 10.1007/978-1-4615-0077-3_7 12908583

[B21] Nagai K. Suda T. Kawasaki K. Mathuura S. (1986). Action of carnosine and beta-alanine on wound-healing. Surgery 100, 815–821. 3095942

[B22] Ni Y. Qiu Y. Jiang W. Suttlemyre K. Su M. Zhang W.. (2012). ADAP-GC 2.0: deconvolution of coeluting metabolites from GC/TOF-MS data for metabolomics studies. Anal. Chem. 84, 6619–6629. 10.1021/ac300898h 22747237

[B23] Nicholson J. K. Lindon J. C. (2008). Systems biology - metabonomics. Nature 455, 1054–1056. 10.1038/4551054a 18948945

[B24] Oja S. S. Saransaari P. (2007). Pharmacology of taurine. Proc. West. Pharmacol. Soc. 50, 8–15. 10.1007/978-0-387-30373-4_8 18605222

[B25] Patti G. J. Yanes O. Siuzdak G. (2012). Metabolomics: the apogee of the omics trilogy. Nat. Rev. Mol. Cell Biol. 13, 263–269. 10.1038/nrm3314 22436749 PMC3682684

[B26] Qiu Y. Cai G. Su M. Chen T. Zheng X. Xu Y.. (2009). Serum metabolite profiling of human colorectal cancer using GC-TOFMS and UPLC-QTOFMS. J. Proteome Res. 8, 4844–4850. 10.1021/pr9004162 19678709

[B27] Rushworth G. F. Megson I. L. (2014). Existing and potential therapeutic uses for N-acetylcysteine: the need for conversion to intracellular glutathione for antioxidant benefits. Pharmacol. Ther. 141, 150–159. 10.1016/j.pharmthera.2013.09.006 24080471

[B28] Sun J. C. Shannon M. Ando Y. Schnackenberg L. K. Khan N. A. Portilla D.. (2012). Serum metabolomic profiles from patients with acute kidney injury: a pilot study. J. Chromatogr. B Anal. Technol. Biomed. Life Sci. 893, 107–113. 10.1016/j.jchromb.2012.02.042 22429878 PMC3325145

[B29] Wei Q. Q. Xiao X. Fogle P. Dong Z. (2014). Changes in metabolic profiles during acute kidney injury and recovery following ischemia/reperfusion. PLoS ONE 9:e106647. 10.1371/journal.pone.0106647 25191961 PMC4156324

[B30] Weiss R. H. Kim K. M. (2012). Metabolomics in the study of kidney diseases. Nat. Rev. Nephrol. 8, 22–33. 10.1038/nrneph.2011.152 22025087

[B31] Wingenfeld P. Minor T. Gehrmann U. Strubind S. Isselhard W. Michalk D. (1995). Hypoxic cellular deterioration and its prevention by the amino-acid taurine in a transplantation model with renal tubular cells (Llc-Pk1). In Vitro Cell. Dev. Biol. Anim. 31, 483–486. 10.1007/BF02634022 8528493

